# Long-term efficacy of transanal local excision versus total mesorectal excision after neoadjuvant treatment for rectal cancer: A meta-analysis

**DOI:** 10.1371/journal.pone.0294510

**Published:** 2023-11-20

**Authors:** Yihui Lei, Li Lin, Qiming Shao, Weiping Chen, Guoyan Liu

**Affiliations:** 1 The School of Clinical Medical, Fujian Medical University, Fuzhou, Fujian, China; 2 Department of Gastrointestinal Surgery, Zhongshan Hospital of Xiamen University, School of Medicine, Xiamen University, Xiamen, Fujian, China; 3 Institute of Gastrointestinal Oncology, Medical College of Xiamen University, Xiamen, Fujian, China; AORN Cardarelli: Azienda Ospedaliera di Rilievo Nazionale Antonio Cardarelli, ITALY

## Abstract

**Aim:**

The purpose of this meta-analysis is to compare the long-term efficacy of transanal local excision (TLE) versus total mesorectal excision (TME) following neoadjuvant therapy for rectal cancer.

**Method:**

The Web of Science, Pubmed, Medline, Embase, and the Cochrane Library were systematically searched for correlational research. The Newcastle-Ottawa Scale and the Cochrane risk of bias tool were used to assess the quality of cohort studies (CSs) and randomized controlled trials (RCTs), respectively. Statistically analyzed using RevMan5.4.

**Result:**

A total of 13 studies, including 3 randomized controlled trials (RCTs) and 10 cohort studies (CSs), involving 1402 patients, were included in the analysis. Of these, 570 patients (40.66%) underwent TLE, while 832 patients (59.34%) underwent TME. In the meta-analysis of CSs, no significant difference was observed between the TLE group and TME group regarding 5-year overall survival (OS) and 5-year disease-free survival (DFS) (P > 0.05). However, the TLE group had a higher rates of local recurrence (LR) [risk ratio (RR) = 1.93, 95%CI (1.18, 3.14), P = 0.008] and a lower rates of 5-years local recurrence-free survival (LRFS) [hazard ratio (HR) = 2.79, 95%CI (1.04, 7.50), P = 0.04] compared to the TME group. In the meta-analysis of RCTs, there was no significant difference observed between the TLE group and TME group in terms of LR, 5-year OS, 5-year DFS, and 5-year disease-specific survival (P > 0.05).

**Conclusion:**

After undergoing neoadjuvant therapy, TLE may provide comparable 5-year OS and DFS to TME for rectal cancer. However, neoadjuvant therapy followed by TLE may has a higher LR and lower 5-year LRFS compared to neoadjuvant therapy followed by TME, so patients should be carefully selected. Neoadjuvant therapy followed by TLE may be a suitable option for patients who prioritize postoperative quality of life. However, the effectiveness of this approach requires further research to draw a definitive conclusion.

## 1. Introduction

Since its introduction in 1982 by Heald et al., total mesorectal excision (TME) surgery has become the established standard for the curative treatment of mid-to-low rectal cancer. This surgical approach has markedly reduced the incidence of local recurrence (LR) compared to traditional surgery and has effectively improved the survival rates of patients [1–4]. Despite these benefits, however, the occurrence of complications such as anastomotic leakage [5, 6], low anterior resection syndrome [7, 8], male urogenital and sexual dysfunction [9, 10], and permanent stoma [11] remains high after TME, leading to a significant decline in quality of life for some patients. This problem still needs to be addressed. Local excision surgeries for rectal cancer include transanal local excision (TLE), Kraske procedure, and Mason procedure. TLE can be further divided into traditional transanal excision, transanal endoscopic microsurgery (TEM), and transanal minimally invasive surgery (TAMIS). Due to the high incidence of rectal skin fistula after Kraske procedure [12], and the tendency for anal incontinence after Mason procedure and the fact that most relevant surgical indications have been replaced by TEM [13–15], these two surgical methods are currently less commonly used in local resection surgery for rectal cancer. TLE is a surgical technique that utilizes the natural cavity for operation, leading to smaller trauma. In comparison to TME, TLE is associated with a lower incidence of surgical complications and a better postoperative quality of life [16–18]. However, LR after TLE in rectal cancer patients is known to be high, even for those with early rectal cancer [19–22]. According to reports, only rectal cancer patients with low-risk pT1 rectal cancer who undergo TLE have a lower LR rates and significant postoperative survival rates [23]. Nonetheless, the use of neoadjuvant therapy has been found to significantly reduce postoperative LR rates in rectal cancer patients [24, 25]. This finding suggests that performing TLE after neoadjuvant therapy may be a feasible strategy for the treatment of rectal cancer. Therefore, we conducted a meta-analysis of the long-term efficacy of TLE versus TME after neoadjuvant treatment for rectal cancer, providing a basis for evaluating its effectiveness.

## 2. Methods

This paper followed the guidelines of the Preferred Reporting Items for Systematic Reviews and Meta-Analysis (PRISMA) [26] and assessing the methodological quality of systematic reviews (AMSTAR2) guidelines [27]. It was registered on the PROSPERO database CRD42023405862.

### 2.1 Literature search

An electronic search was done by two independent researchers (LYH and LL) from the databases of Web of Science, Pubmed, Medline, Embase, and the Cochrane Library to search for the relevant studies which were published from inception of these databases to March 1st, 2023. No restrictions were entered for the search. A literature search was performed using the following index terms: “rectal cancer”, “total mesorectal excision”, “transanal endoscopic microsurgery”, “transanal minimally invasive surgery”, “transanal local excision” and “local transanal excision”.

### 2.2 Inclusion criteria

Inclusion criteria:(1) Only published English cohort studies (CSs) or randomized controlled trials (RCTs) will be included. (2) The diagnosis of rectal cancer should include pathological examination, clinical evaluation, colonoscopy, and one or more imaging examinations. (3) No distant metastases. (4) The outcome indicators should include at least one of the following: LR, overall survival (OS), disease-free survival (DFS), local recurrence-free survival (LRFS), and disease-specific survival (DSS). (5) The experimental group underwent TLE, including TEM, TAMIS, and traditional transanal surgery, with complete local excision of the lesion. The control group underwent TME, including laparoscopic, open, and robot-assisted rectal cancer resection surgeries. (6) Both the experimental and control groups received at least one type of neoadjuvant therapy, either radiotherapy or chemotherapy. (7) The total number of cases included in the study should be greater than or equal to 20.(8) Median follow-up time≥ 36 months.

### 2.3 Exclusion criteria

Exclusion criteria: (1) Patients with recurrent rectal cancer. (2) Basic research such as animal experiments. (3) Lack of sufficient data which we are interesting or cannot be calculated from the article data; (4) Duplicate data or repeat analysis. (5) The full text is not available. (6) Conference abstract.

### 2.4 Data collection

The data were extracted independently by 2 investigators (LYH and LL) and discrepancies were resolved in consultation with a third author (LGY). In cases where the same study population was reported in multiple research reports, the study with a greater sample size was chosen. If a study performed propensity score matching on two groups of patients, only the data from the propensity-score matched cohort was included. From each study the following information were collected: (1) author; (2) time of publication of the literature; (3) the country of study; (4) number of patients; (5) age of patients; (6) median follow-up time; (7) tumor clinical stage; (8) tumor pathological T staging; (9) histological grade; (10) tumor response after neoadjuvant therapy; (11) preoperative examination method; (12) surgical margin; (13) LR; (14) LRFS; (15) OS; (16) DFS; (17) DSS. Moreover, the HRs and 95% CIs for each endpoint were extracted.

### 2.5 Quality assessment

Two researchers independently evaluated all included studies. The Newcastle-Ottawa Scale and Review Manager (RevMan) computer program (version 5.4. Copenhagen, Denmark: The Nordic Cochrane Centre, The Cochrane Collaboration, 2020) were used to assess the quality of CSs and RCTs, respectively. The CSs were evaluated based on the selection of study population, comparability between TLE and TME groups, and outcome assessment. The RCTs were evaluated based on several aspects, including random sequence generation, allocation concealment, blinding of participants and personnel, blinding of outcome assessment, incomplete outcome data, selective reporting, other bias. Additionally, funnel plot for LR in CSs was analyzed to evaluate publication bias.

### 2.6 Statistical analysis

All statistical analyses were conducted using Revman Manager 5.4, which was provided by the Cochrane Collaboration. Treatment outcomes were expressed as risk ratios (RR) or hazard ratio (HR) and calculated from the raw data extracted from each study. Heterogeneity among the included studies was evaluated using the I^2^ values. It was considered indicative of low, moderate and high heterogeneity when I^2^ statistic ≤ 25%, 25% < I^2^ statistic < 50%, and I^2^ statistic ≥ 50%, respectively. An I^2^ statistic ≤ 50% showed no significant heterogeneity, and the fixed-effects model was used. If there was statistical heterogeneity among the results of each study, meta-regression analysis and subgroup analyses were applied to investigate factors for heterogeneity. After excluding the influence of obvious clinical heterogeneity, the random effect model was applied. For meta-analyses that include more than 5 studies, we conducted sensitivity analysis by sequentially removing one study or changing the meta-analysis model to evaluate the stability of the results. Differences were considered statistically significant at a P-value of ≤ 0.05.

## 3. Result

### 3.1 Selection of studies

**Fig 1:**
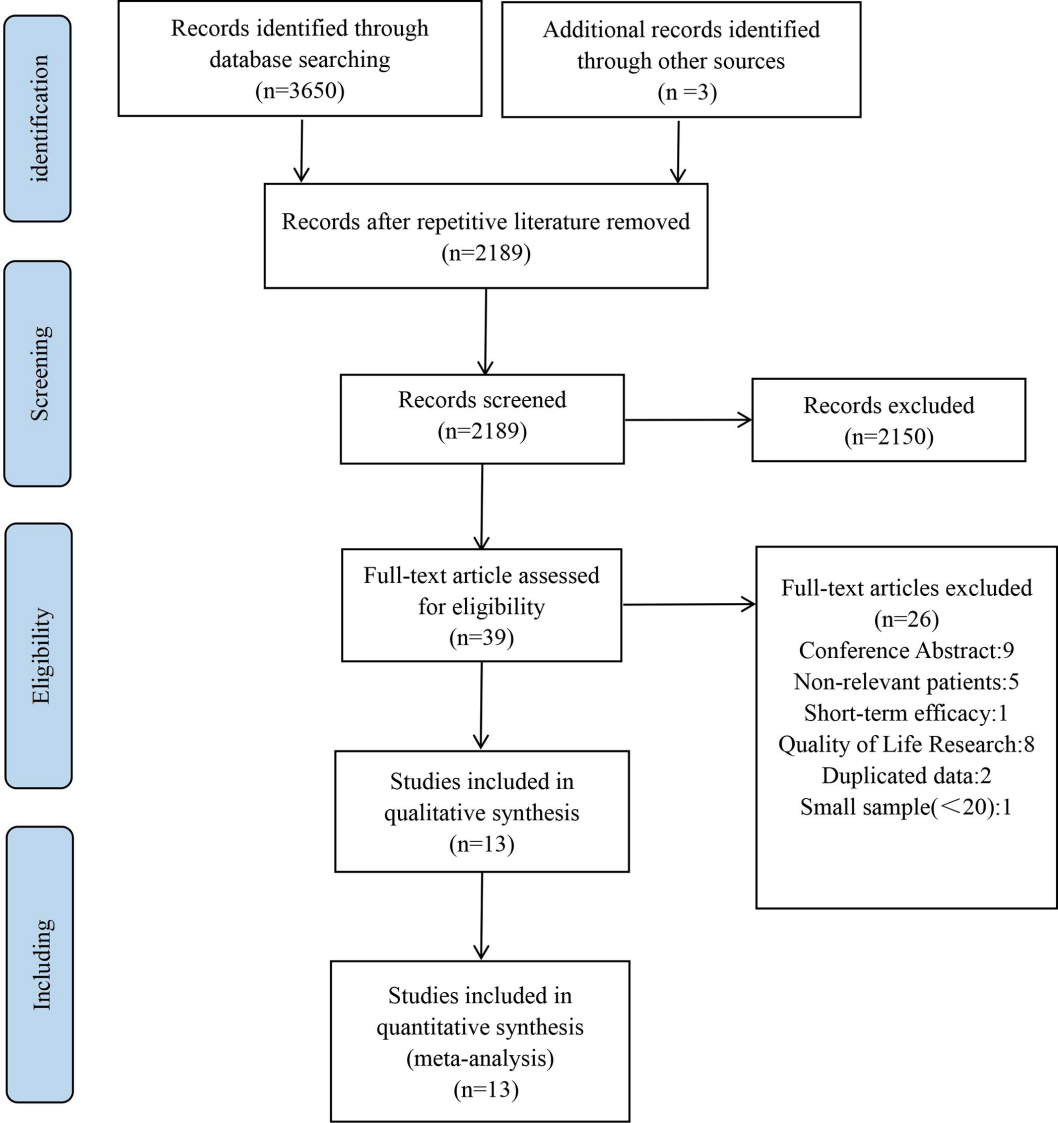
Flow chart of literature search and study selection.

A total of 3653 relevant publications were identified on primary literature search. After removing the duplicates, 2189 articles were identified as eligible, of which 2150 were eliminated after reading of the title and abstract. A total of 39 studies were assessed for eligibility with full-text. Based on the exclusion and inclusion criteria, twenty-six of them were excluded because 9 was conference abstract, 5 reported non-relevant patients, 1 analyzed short-term efficacy, 2 were duplicate data, 8 was quality of life research and 1 has small sample. Lastly, 13 literatures embracing 1402 patients met the inclusion criteria, which were included for extracting needed data (**Fig 1)**.

### 3.2 Characteristics of included studies

Characteristics of the included studies are shown in (**Tables 1** and **2**). Of all 13 studies, ten were CSs (including six propensity score matching analyses) [28–37], three were RCTs [38–40], ten CSs and three RCTs reported LR, four CSs reported LRFS, six CSs and two RCTs reported OS, six CSs and three RCTs reported DFS, two RCTs reported DSS. This study included 1402patients, of which 570 patients (40.66%) underwent TLE and 832 patients (59.34%) underwent TME.

**Table 1:** Characteristics of studies included in meta-analysis.

Study	Country	Study design	Study groups		Sex, M:F		Age(y)		Pretreatment T category		Pretreatment N category		Outcome	Median follow-up time (month)	
			TLE	TME	TLE	TME	TLE	TME	TLE	TME	TLE	TME		TLE	TME
Shin et al. [37], 2017	Korea	PSMA	55	55	34:21	31:24	61 (36–81)	60 (36–81)	T3:55	T3:55	N-:47 N+:8	N-:48 N+:7	OS, DFS, LRFS, LR	45.0	45.0
Shin et al. [36], 2018	Korea	PSMA	48	48	29:19	27:21	64 (36–81)	62 (39–78)	T2:11 T3:37	T2:10 T3:38	N1:27 N2:21	N1:31 N2:17	OS, DFS, LRFS, LR	54.0	54.0
Rizzo et al. [35], 2022	Italy	CS	35	58	22:13	37:21	61.2±3.5	60.4±3.5	T2:4 T3:31	T2:3 T3:55	N0:24 N1:11	N0:33 N1:25	OS, DFS, LR	62.0	72.0
Oh et al. [34], 2018	Korea	PSMA	45	45	29:16	25:20	NR	NR	T1~2:21 T3:24	T1~2:14 T3:31	N-:29 N+:16	N-:19 N+:26	OS、LR	60.1	58.5
Calmels et al. [30], 2020	France	PSMA	39	71	22:17	47:24	68±12	61±10	T3~4:39	T3~4:71	NR	NR	DFS, LR	47.0	67.0
Bushati et al. [29], 2019	Italy	PSMA	51	51	34:17	32:19	66 (36–85)	67 (43–84)	T2:19 T3:23 T4:9	T2:6 T3:35 T4:9	N-:21 N+:29	N-:5 N+:45	OS, DFS, LRFS, LR	61.0	64.0
Jung et al. [32], 2016	Korea	PSMA	42	42	24:18	22:20	60.6±9.2	59.0±8.6	T2:20 T3~4:22	T2:17 T3~4:24	N-:18 N+:24	N-:17 N+:25	OS, DFS, LR	53.4	58.0
Belluco et al. [28], 2016	Italy	CS	47	179	NR	NR	NR	NR	NR	NR	NR	NR	LR	48.0	48.0
Marks et al. [33], 2013	America	CS	49	112	NR	NR	67.8(29–90)	61.0(22~85)	T1:2 T2:25 T3:20 T4:2	T1:1 T2:23 T3:88 T4:0	NR	NR	LR, LRFS	36.3	38.6
Caricato et al. [31], 2006	Italy	CS	8	22	NR	NR	NR	NR	T2:3 T3:4 T4:1	T2:5 T313 T4:4	N-:6 N+:2	N-:4 N+:18	LR	37.0	48.0
Rullier et al. [40], 2020	France	RCT	74	71	24:50	28:43	61 (35–84)	64 (40–88)	T2:41 T3:33	T2:36 T3:35	N0:42 N1:32	N0:48 N1:23	DFS, LR, DSS	60.0	60.0
Lezoche et al. [39], 2012	Italy	RCT	50	50	30:20	34:16	66 (58–70)	66 (60–69)	T2:50	T2:50	N0:50	N0:50	DFS, DSS, LR	115.2	115.2
Bach et al. [38], 2021	England	RCT	27	28	19::8	17:11	65 (52–79)	65 (49–83)	Tx:1 T1:10 T2:16	Tx:2 T1:5 T2:21	N0:27	N0:28	OS, DFS, LR	51.3	51.3

**Note.** M: male; F: female; CS: cohort study; TLE: transanal local excision; TME: total mesorectal excision; PSMA: propensity score matching analysis; LR: local recurrence; OS: overall survival; DFS: disease-free survival; LRFS: local recurrence-free survival; CS: cohort study; NR: no reported; RCT: randomized controlled trial; DSS: disease-specific survival.

**Table 2:** Characteristics of TLE group in included studies.

Study		Tumor response	Preoperative evaluation method		Composition of preoperative	Histological grade	Surgical margin	Adverse pathological features	Salvage surgery	adjuvant therapy	pT staging	Resection margin
Shin et al. [37], 2017		cCR: without abnormal echoic lesion by EUS or tumor signal by T2- or diffusion-weighted MRI. ncCR: only a small residual lesion with uncertain viability was visible in each modality.	TUS, MRI		Patients who achieved cCR or a small portion of patients who refused or were unable to undergo TME surgery.	WD: 12 (21.8%) MD: 42 (76.4%) PD: 1 (1.8%)	≥1cm	NR	LR: 2(3.6%) DM: 1(1.8%) APF: NR	29(52.7%)	ypT0: 36 (65.5%) ypT1: 9 (16.4%)	NR
Shin et al. [36], 2018		cCR: without abnormal echoic lesion by EUS or tumor signal by T2- or diffusion-weighted MRI. ncCR: only a small residual lesion with uncertain viability was visible in each modality.	Endoscopy, EUS, and MRI		Patients who achieved cCR or a small portion of patients who refused or were unable to undergo TME surgery.	WD: 12 (25.0%) MD: 35 (72.9%) PD: 1 (2.1%)	≥1cm	NR	LR:0 DM: 3(6.2%) APF: NR	27(56.3%)	ypT0: 25 (52.1%) ypT1: 12 (25.0%)	NR
Rizzo et al. [35], 2022	cCR: no palpable mass at DRE, no residual tumor or a white scar at proctoscopy, and absence of positive lymph nodes on MRI. ncCR: only a superficial ulcer smaller than 2 cm at proctoscopy.	DRE, MRI, Endoscopy		NR	NR	NR	ypT>1or ypT1 with TRG>2	0	NR	ypT0: 27 (77.1%) ypT1: 8 (22.9%)	R0: 35(100%)
Oh et al. [34], 2018		cCR: absence of residual mass or ulceration on DRE, colonoscopy, EUS, or APCT; without tumor signal by diffusion-weighted MRI and low signal intensity of the apparent diffusion coefficient map in MRI; absence of significant focal discrete uptake on the rectal wall in PET; or no increase of serum CEA levels.	Endoscopy, EUS, PET CT scan, MRI		cCR:11 (24.4%) Others:34 (75.6%)	WD/MD: 41 (91.1%) PD:4 (8.9%)	NR	NR	NR	NR	NR	NR
Calmels et al. [30], 2020		CTR:(1) no mucosal abnormality observed in clinical rectal examination or only small residual scars (< 2 cm in diameter) and without any evidence of macroscopic residual rectal tumor and (2) absence of residual tumor and positive lymph nodes on MRI.	Endoscopy, CT scan, MRI		Patients achieved CTR or a small portion of patients who have high-risk patients with severe comorbidities.	NR	1cm	yp ≥ T2, R1, LVI	LR:0 APF: 3(7.7%)	NR	ypT0~1: 28(71.8%)	R0: 36(92.3%) R1: 3(7.7%)
Bushati et al. [29], 2019		cCR: no palpable mass at DRE, no residual tumor or a white scar at proctoscopy, and absence of positive lymph nodes on MRI. ncCR: only a superficial ulcer smaller than 2 cm at proctoscopy.		DRE, MRI, Endoscopy	cCR or ncCR: 51(100%)	NR	≥5mm	ypT2-3, TRG3–5, R1, LVI or PD	LR: 4(7.8%) DM: 1(2.0%) APF: 3(5.9%)	NR	ypT0: 35 (68.6%) ypT1: 6 (11.8%)	NR
Jung et al. [32], 2016		NR	MRI, TUS, Endoscopy		Patients of good clinical response or a small portion of patients who refused or were unable to undergo TME surgery.	WD: 18 (42.8%) MD: 23 (54.8%) PD: 1 (2.4%)	≥1cm	NR	LR: 2(4.8%) DM:0 APF: NR	15(35.7%)	ypT0: 25 (59.5%) ypTis: 6 (14.3%) ypT1: 11 (26.2%)	NR
Belluco et al. [28], 2016		NR	EUS, MRI		Patients with a major clinical response or had medical comorbidity or refusal of APR.	NR	NR	NR	NR	NR	NR	NR
Marks et al. [33], 2013		Complete response: no residual tumor, surface abnormality, or mural involvement. Good response:75% or greater reduction in tumor size, and induration. Moderate response:25–75% reduction in tumor size and induration. Minimal response:<25% reduction in tumor size or induration.	NR		Patients who refused radical surgery or if there was disease regression to within the rectal wall of<3 cm.	NR	≥1 cm	ypT3 or N+	NR	NR	ypT0: 11 (22.4%) ypT1: 3 (6.1%)	R0: 49(100%)
Caricato et al. [31], 2006		Significant clinical response: no mesorectal involvement on ultrasound or CT scan; no lymph node involvement. Loco-regional lymph nodes were defined as positive if they appeared larger than 1 cm in diameter on ultrasound or CT scan; no fixity at digital examination; ulcer smaller than 2 cm at proctoscopy.	CT scan, TUS, Endoscopy, Barium enema		Significant clinical response:8(100%)	NR	NR	NR	LR: 1(12.5%) DM:0 APF: 1(12.5%)	NR	ypT0: 3 (37.5%) ypT1: 1 (12.5%)	NR
Rullier et al. [40], 2020		cCR or ncCR: tumor scar of 2 cm or less, with no vegetative component and no significant hollow or deep infiltration into the muscular layer.	MRI		cCR or ncCR: 74(100%)	NR	1cm	ypT2–3 or R1	LR: NR DM: NR APF: 25(33.8%) Others: 1(1.4%)	3(4%)	ypT0~1: 41(55.4%)	NR
Lezoche et al. [39], 2012		Responders: tumor mass reduction at least 50 per cent. Low or non-responders: tumor mass reduction less than 50 per cent.	CT, MRI, EUS		NR	WD/MD: 50(100%)	≥1cm	NR	NR	0	ypT0: 14 (28%) ypT1: 12 (24%)	R0: 50(100%)
Bach et al. [38], 2021		NR	NR		NR	WD/MD: 17 (63.0%) PD:2(7.4%)	1cm	Tumor diameter>30 mm, R1, PD, LVI, ypT≥3	LR: NR DM: NR APF: 7(26.0%)	NR	ypT0: 7 (26.0%) ypT1: 6 (22.2%)	R0: 23(85.1%) R1: 3(11.1%)

**Note**. cCR: clinical complete response; EUS: endoscopic ultrasonography; MRI: magnetic resonance imaging; ncCR: near clinical complete response; TUS: transanal ultrasonography; TME: total mesorectal excision; WD: well differentiated; MD: moderately differentiated; PD: poorly differentiated; NR: no reported; LR: local recurrence; DM: distant metastasis; APF: adverse pathological features; DRE: digital rectal examination; TRG: tumor regression grade; R: rescection; APCT: abdominopelvic computed tomography; CEA: carcinoembryonic antigen; PET: positron emission tomography; CT: computed tomography; CTR: complete tumor response; LVI: lymphovascular invasion; N+: Positive lymph node.

### 3.3 Risk of bias in the included studies

**Fig 2:**
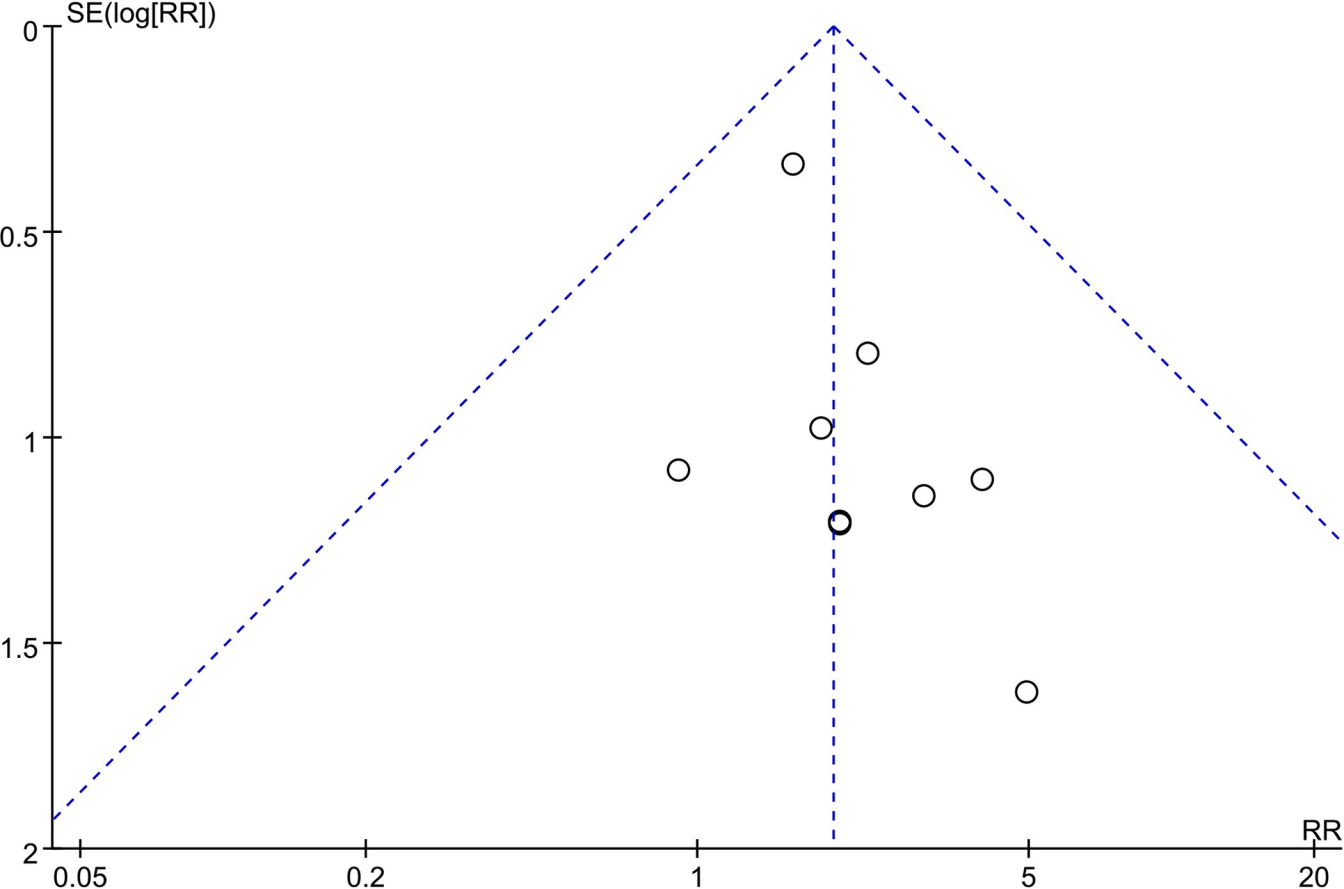
Funnel plot for local recurrence in cohort studies.

(**Tables 3** and **4**) show the risk of bias for the three RCTs and ten CSs, respectively. All RCTs [38–40] presented a high performance bias, because trialists need to perform salvage surgery on TLE group patients with adverse pathological results or recurrence, so blinding of trial personnel is not feasible. Morever, in the studies by Rullier et al. [40] and Bach et al. [38], TLE group included patients who underwent salvage surgery, while in the study by Lezoche et al. [39], there was no report on the relevant information regarding salvage surgery in TLE group, hence all RCTs were tagged with unclear risk of other bias. All 10 CSs had a score of ≥7, indicating a low risk of bias. Among them, 9 CSs [28–30, 32–37] were rated as having incomplete follow-up due to their failure to describe the attrition rate, while 3 studies [31, 33, 35] were deducted points in terms of comparability between groups due to a lack of control for relevant confounding factors. The risk of publication bias was considered low, since the funnel plot for LR in CSs did not show asymmetries (**Fig 2**).

**Table 3:** Evaluation of the methodological quality of the randomized controlled trials using the Cochrane tool.

	Random sequence generation	Allocation concealment	Blinding of participants and personnel	Blinding of outcome assessment	Incomplete outcome data	Selective reporting	Other bias
Rullier [40], 2020	low	low	high	low	low	low	Unclear
Lezoche [39], 2012	low	low	high	low	low	low	Unclear
Bach [38], 2021	low	low	high	low	low	low	Unclear

**Table 4:** Newcastle-Ottawa Scale of the included studies.

Study	Selection				Comparability of cohorts on the basis of the design or analysis	Outcome			Total score
	Representativeness of the exposed cohort	Selection of the non- exposed cohort	Ascertain-ment of exposure	Demonstration that outcome of interest was not present at start of study		Assessment of outcome	Was follow-up long enough for outcomes to occur	Adequacy of follow up of cohorts	
Shin et al. [37], 2017	1	1	1	1	2	1	1	0	8
Shin et al. [36], 2018	1	1	1	1	2	1	1	0	8
Rizzo et al. [35], 2022	1	1	1	1	1	1	1	0	7
Oh et al. [34], 2018	1	1	1	1	2	1	1	0	8
Calmels et al. [30], 2020	1	1	1	1	2	1	1	0	8
Bushati et al. [29], 2019	1	1	1	1	2	1	1	0	8
Jung et al. [32], 2016	1	1	1	1	2	1	1	0	8
Belluco et al. [28], 2016	1	1	1	1	2	1	1	0	8
Marks et al. [33], 2013	1	1	1	1	1	1	1	0	7
Caricato et al. [31], 2006	1	1	1	1	1	1	1	1	8

### 3.4 Meta-analysis results of cohort studies

#### LR

**Fig 3:**
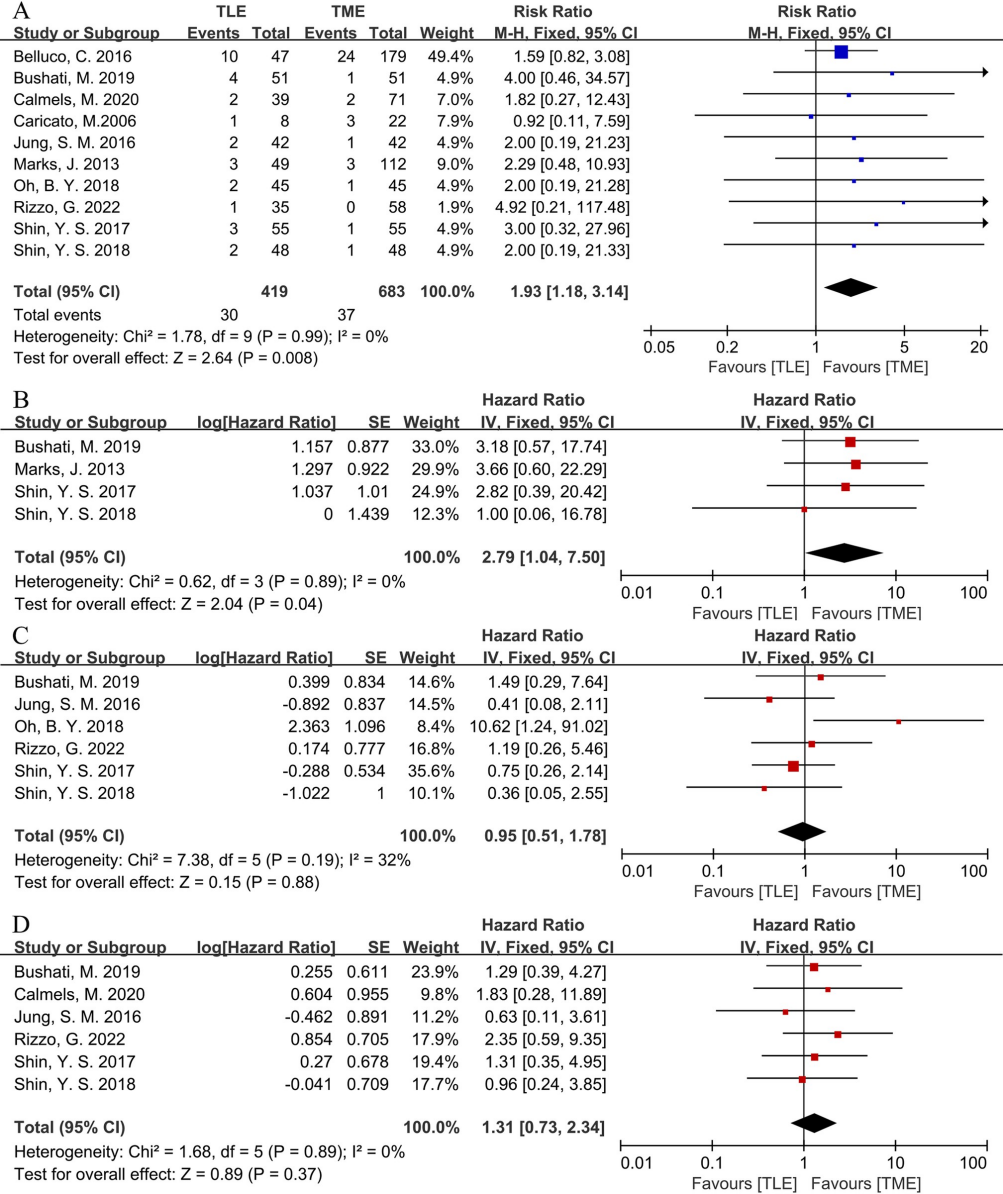
Forest plots of survival outcomes between TLE group and TME group in CSs. (A) LR. (B) 5-year LRFS. (C) 5-year OS. (D) 5-year DFS.

Ten studies reported LR [28–37]. Heterogeneity across the studies was not significant (P = 0.99, I^2^ = 0%). Therefore, we conducted a meta-analysis using a fixed-effects model. Significant difference was found with respect to LR between the 2 groups [RR = 1.93, 95%CI (1.18, 3.14), P = 0.008] (**Fig 3A**), suggesting a correlation between TLE and increased LR. Deleting any single study or converting to a random effects model did not affect the results of this study, indicating that the fixed effects model’s calculated results are stable and reliable.

#### 5-year LRFS

Four studies reported 5-year LRFS [29, 33, 36, 37]. Heterogeneity across the studies was not significant (P = 0.89, I^2^ = 0%). Therefore, we conducted a meta-analysis using a fixed-effects model. Significant difference was found with respect to 5-year LRFS between the 2 groups [HR = 2.79, 95%CI (1.04, 7.50), P = 0.04] (**Fig 3B**), suggesting a correlation between TLE and lower 5-year LRFS.

#### 5-year OS

Six studies reported 5-year OS [29, 32, 34–37]. Heterogeneity across the studies was not significant (P = 0.19, I^2^ = 32%). Therefore, we conducted a meta-analysis using a fixed-effects model. No significant difference was found with respect to 5-year OS between the 2 groups [HR = 0.95, 95%CI (0.51, 1.78), P = 0.88] (**Fig 3C**). Deleting any single study or converting to a random effects model did not affect the results of this study, indicating that the fixed effects model’s calculated results are stable and reliable.

#### 5-year DFS

Six studies reported 5-year DFS [29, 30, 32, 35–37]. Heterogeneity across the studies was not significant (P = 0.89, I^2^ = 0%). Therefore, we conducted a meta-analysis using a fixed-effects model. No significant difference was found with respect to 5-year DFS between the 2 groups [HR = 1.31, 95%CI (0.73, 2.34), P = 0.37] (**Fig 3D**). Deleting any single study or converting to a random effects model did not affect the results of this study, indicating that the fixed effects model’s calculated results are stable and reliable.

### 3.5 Meta-analysis results of randomized controlled studies

#### LR

**Fig 4:**
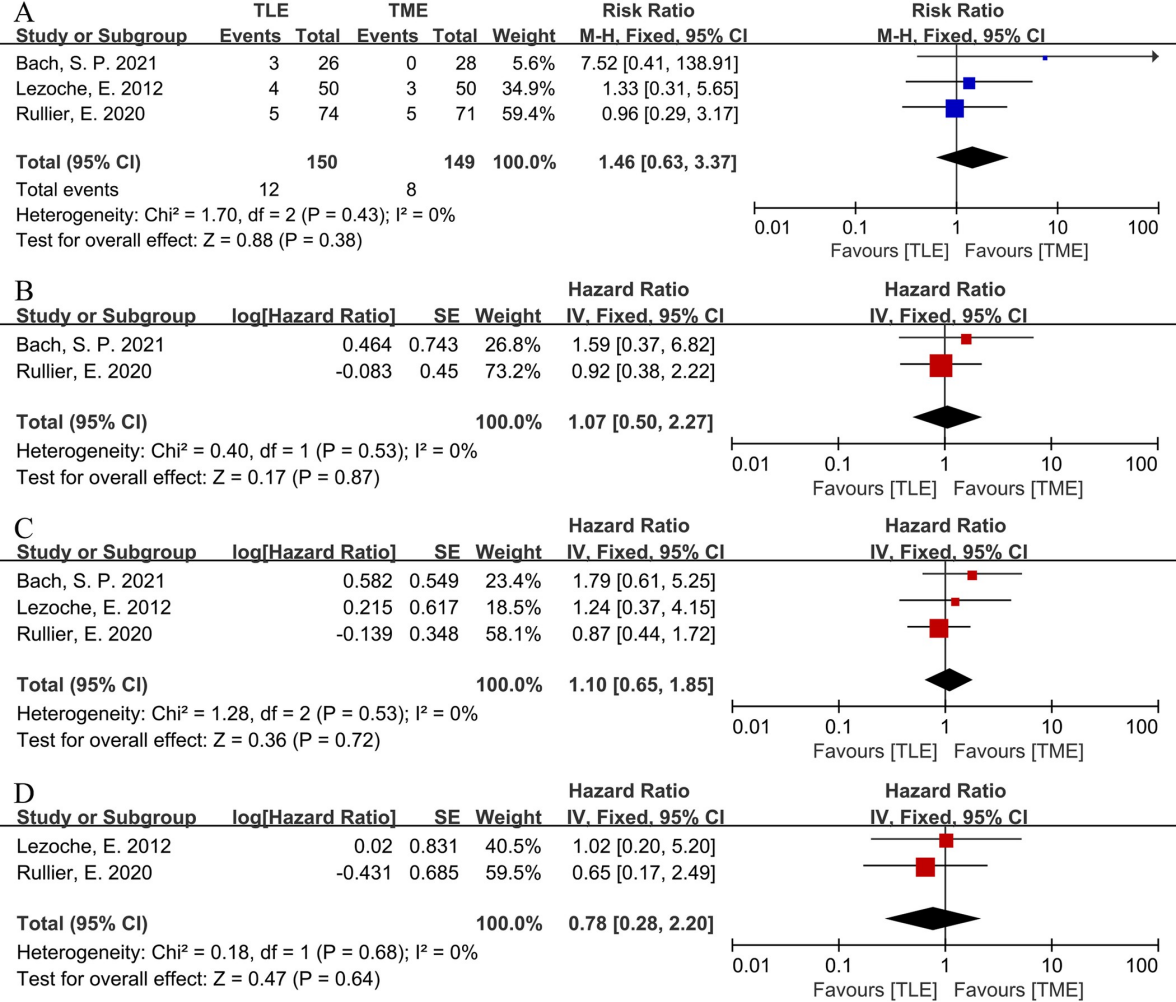
Forest plots of survival outcomes between TLE group and TME group in RCTs. (A) LR. (B) 5-year OS. (C) 5-year DFS. (D) 5-year DSS.

Three studies reported LR [38–40]. Heterogeneity across the studies was not significant (P = 0.43, I^2^ = 0%). Therefore, we conducted a meta-analysis using a fixed-effects model. No significant difference was found with respect to LR between the 2 groups [RR = 1.46, 95%CI (0.63, 3.37), P = 0.38] (**Fig 4A**).

#### 5-year OS

Two studies reported 5-year OS [38, 40]. Heterogeneity across the studies was not significant (P = 0.53, I^2^ = 0%). Therefore, we conducted a meta-analysis using a fixed-effects model. No significant difference was found with respect to 5-year OS between the 2 groups [HR = 1.07, 95%CI (0.50, 2.27), P = 0.87] (**Fig 4B**).

#### 5-year DFS

Three studies reported 5-year DFS [38–40]. Heterogeneity across the studies was not significant (P = 0.53, I^2^ = 0%). Therefore, we conducted a meta-analysis using a fixed-effects model. No significant difference was found with respect to 5-year DFS between the 2 groups [HR = 1.10, 95%CI (0.65, 1.85), P = 0.72] (**Fig 4C**).

#### 5-year DSS

Two studies reported 5-year DSS [39, 40]. Heterogeneity across the studies was not significant (P = 0.68, I^2^ = 0%). Therefore, we conducted a meta-analysis using a fixed-effects model. No significant difference was found with respect to 5-year DSS between the 2 groups [HR = 0.78, 95%CI (0.28, 2.20), P = 0.64] (**Fig 4D**).

## 4. Discussion

In recent years, there has been controversy surrounding the effectiveness of local excision after neoadjuvant chemoradiotherapy for rectal cancer.

In 2016, Hallam et al. [41] reported a meta-analysis of 1068 rectal cancer patients who underwent neoadjuvant therapy and local excision surgery. The results showed that the pooled crude rate of LR was 11.8% in cT2 tumors, 13.3% in cT3 tumors, 4% in ypT0 tumors, 12.1% in ypT1 tumors, 23.6% in ypT2 tumors. Pooled median DFS for ypT0 tumors were 95.0%, while for ypT1 tumors or higher were 68.0%. This indicates that only with complete pathological response, can the local excision after neoadjuvant therapy achieve good efficacy. A systematic review by Peltrini et al. [42] found that TEM after neoadjuvant chemoradiotherapy in cT2 stage rectal cancer patients had favorable outcomes, with a pooled 5-year DFS of 91.3%, a 5-year OS of 72.6%, and a 4% of LR rate. Shaikh et al. [43] reported a meta-analysis comparing the efficacy of local excision or radical resection in rectal cancer patients without distant metastasis after neoadjuvant therapy. The study did not impose specific restrictions on the tumor characteristics of the included patients. Ultimately, the analysis found no significant differences between the two methods in terms of LR, OS, and DFS. Similarly, Ahmad et al. [44] reported a meta-analysis comparing the efficacy of TEM or TME in early-stage rectal cancer patients with moderate or high tumor differentiation, and found no significant difference in LR between the two groups.

The meta-analysis results of CSs in this paper indicated that while patients with rectal cancer who underwent TLE after neoadjuvant therapy may have provided comparable 5-year OS and 5-year DFS to those who underwent TME surgery, the LR in the TLE group was significantly higher and the 5-year LRFS was significantly lower compared to the TME group. However, the meta-analysis of RCTs showed no significant statistical difference in LR between the TLE and TME groups. In addition to the small sample size in RCTs, the reason for the difference in results may also be related to the composition of TLE group patients in both RCTs and CSs, including the tumor stage before neoadjuvant therapy, tumor stage after neoadjuvant therapy, tumor pathological characteristics, and the proportion of salvage surgery performed due to adverse pathological factors. Apart from surgical methods, patients with stage II/III/IV rectal cancer based on TMN staging have been proven to be a risk factor for LR after surgery [45, 46]. The 2022 NCCN guidelines recommend that TLE only be performed for early rectal cancer patients with T1N0 staging who meet the following preoperative criteria: the tumor size should be less than 30% of the circumference of the rectum, the tumor diameter should be < 3cm, surgical margin > 3mm, moderately or well differentiated tumor, located within 8 cm from the anal margin, and no evidence of lymph node metastasis [47]. In recent years, most patients who underwent TLE in studies regarding rectal cancer after neoadjuvant therapy achieved clinical complete remission (cCR) or near-nCR (ncCR), which is similar to the selection criteria in the NCCN guidelines [29, 30, 33, 36, 37, 40]. However, accurately determining cCR/ncCR has emerged as a formidable challenge. In addition to the definition of cCR/ncCR by the researchers themselves, it is crucial to accurately apply imaging methods for assessment. Currently, there is no consensus on the most accurate method for restaging after neoadjuvant therapy in rectal cancer. However, based on existing studies, a combined examination approach may be a preferable assessment method. Cho et al. [48] reported that the accuracy of predicting ypT0 using a combination of MRI and endoscopy can reach up to 84.55%. Nahas et al. [49] demonstrated an accuracy of 83% in predicting cCR through the combined use of MRI and endoscopy. In a prospective study reported by Maas et al. [50], the utilization of MRI, diffusion-weighted imaging, and endoscopy for predicting cCR demonstrated an impressive accuracy rate of 98%. Furthermore, (18)F-fluorodeoxyglucose positron emission tomography, diffusion-weighted imaging, and dynamic contrast-enhanced magnetic resonance imaging may be considered as superior standalone diagnostic methods [51–53]. A proper preoperative staging can yield significant postoperative outcomes. In a cohort study reported by Bushati et al. [29], rectal cancer patients who achieved cCR/ncCR (defined as no palpable mass at digital rectal examination, no residual tumor or a residual tumor scar of less than 2 cm at proctoscopy, and absence of positive lymph nodes on magnetic resonance imaging) after neoadjuvant therapy were assigned to the TLE group, while the remaining patients were assigned to the TME group. After a median follow-up period of 61 months, no significant statistical differences were observed in 5-year LRFS, 5-year DFS, and 5-year OS between the two groups. Rullier et al. [40] reported a phase III clinical trial involving rectal cancer patients who showed good clinical response after neoadjuvant therapy (defined as a residual tumor scar of 2 cm or less with no vegetative component and no significant hollow or deep infiltration into the muscular layer), and were randomly assigned to either the TLE or TME group. The multicenter trial reported no significant differences in LR, 5-year OS, 5-year DFS, and 5-year cancer-specific mortality between the two groups.

The pathological status after rectal cancer surgery is also related to the LR. Studies showed that rectal cancer patients with lymphovascular invasion, extramural venous invasion, positive margins, serosal involvement, and poorly differentiated tumor cells confirmed by postoperative histopathology had a much higher LR rates than those without [45, 46]. Therefore, a favorable postoperative pathology is crucial for excellent postoperative outcomes. The study reported by Belluco et al. [28] demonstrated that rectal cancer patients with postoperative pathological staging of ypT0 had significantly improved 5-year LRFS, 5-year DSS, and 5-year DFS compared to those no-ypT0. Rullier et al. [40] reported the survival outcomes of patients with ypT0-1 rectal cancer in a phase III clinical trial. The results showed no significant differences in LR, 5-year OS, 5-year DFS, and 5-year cancer-specific mortality between the TME group and the TLE group. In the cohort studies reported by Rizzo et al. [35] and Jung et al. [32], the efficacy of TLE and TME were compared in patients with ypT0-1 rectal cancer. The LR rates in the TLE group were only 2.8% (1/35) and 4.7% (2/42) in Rizzo’s and Jung’s studies, respectively. Additionally, in both studies, there were no significant difference observed between the TLE and TME groups in terms of LR, 5-year OS, and 5-year DFS. For patients who present with adverse pathological features such as positive surgical margins after TLE surgery, NCCN guidelines recommends salvage surgical treatment [47]. Currently, there is a preliminary understanding of the effectiveness of salvage TME surgery after local excision surgery. Chaouch MA et al. [54] demonstrated that compared to the initial TME surgery, salvage TME surgery had similar postoperative morbidity, LR rate, and mortality rate, despite longer operation time and poorer quality of pathological specimens. Nonetheless, due to most of the articles included in the systematic review by Chaouch MA et al. [54] were non-randomized controlled trials, it is still necessary to assess the potential adverse impact of salvage TME surgery on patients’ quality of life. Overall, these studies seem to provide a reliable basis for performing TLE on rectal cancer patients after neoadjuvant therapy in the future.

In the CSs included in this article, eight studies reported the probability of ypT0-1 in the TLE group, which were 80.4% (41/51), 71.8% (28/39), 50% (4/8), 100% (42/42), 28.6% (14/49), 100% (35/35), 77.1% (37/48) and 81.9% (45/55) [29–33, 35–37]. Four studies reported the tumor differentiation degree in the TLE group, with rates of 97.6% (41/42), 91.1% (41/45), 97.9% (47/48), 98.2% (54/55), for well/moderately differentiated tumors, respectively [32, 34, 36, 37]. Three studies reported the proportion of TLE patients undergoing salvage surgery due to adverse pathological features, with the rates of 5.9% (3/51), 7.7% (3/39), and 12.5% (1/8) [29–31]. Three studies reported the preoperative probability of cCR/ncCR in the TLE group, with the rates of 100% (51/51), 100% (8/8), 24.4% (11/45) [29, 31, 34]. In the RCTs included in this article, three studies reported the probability of ypT0-1 in the TLE group, which were 48.1% (13/27), 52.0% (26/50), and 55.4% (41/74) [38–40]. Two studies reported the tumor differentiation degree in the TLE group, with rates of 63.0% (17/27) and 100% (50/50) for well/moderately differentiated tumors, respectively [38, 39]. Two studies reported the proportion of TLE patients undergoing salvage surgery due to adverse pathological features, with the rates of 26.0% (7/27) and 33.8% (25/74) [38, 40]. Only Rullier et al.’s study reported the preoperative cCR/ncCR ratio in the TLE group, which was 100% (74/74) [40]. The remaining CSs and RCTs did not report detailed information on tumor pathology and preoperative patient characteristics in the TLE group.

Therefore, the reasons for the differences between CSs and RCTs in terms of LR may be attributed to the following factors. Firstly, RCTs included only three studies with a small number of patients, which may limit the representativeness of the results. Secondly, the higher proportion of patients with advanced-stage tumors in the TLE group of CSs may result in elevated rates of LR and decreased rates of LRFS. Lastly, RCTs have a higher number of patients undergoing salvage surgery due to adverse pathological factors, and the higher proportion of salvage surgeries in RCTs may have resulted in a lower LR rate.

However, as previously stated, patients with rectal cancer who undergo TME surgery often experience numerous postoperative complications and a diminished quality of life. For those patients who cannot bear the postoperative decline in quality of life, especially those who refuse permanent ostomies, TLE is still a good option. Nonetheless, strict inclusion criteria must still be enforced, and salvage surgery should be performed on patients with adverse pathological features.

This study has significant limitations. Firstly, the TLE group consists of patients with diverse characteristics, lacking consistency in clinical staging before neoadjuvant therapy, clinical staging after neoadjuvant therapy, or postoperative pathological staging. Secondly, different studies employed varied neoadjuvant treatment regimens, and the proportion of patients receiving adjuvant therapy after surgery is unknown. Thirdly, the TLE group includes patients who underwent salvage TME surgery due to local recurrence or adverse pathological findings, which to some extent contributed to improved survival rates. Fourthly, variations in follow-up protocols among studies, including the frequency of follow-up and the methods of examination, may potentially have an impact on postoperative survival rates. Finally, most of the studies included in this meta-analysis were CSs, with only three RCTs, making it difficult to interpret the results accurately. Therefore, further large-scale, high-quality clinical trials are needed to demonstrate the safety of TLE after neoadjuvant radiotherapy and chemotherapy in rectal cancer.

## 5. Conclusion

After undergoing neoadjuvant therapy, TLE may provide comparable 5-year OS and DFS to TME for rectal cancer. However, neoadjuvant therapy followed by TLE has a higher LR and lower 5-year LRFS compared to neoadjuvant therapy followed by TME, so patient selection should be carefully considered. Neoadjuvant therapy followed by TLE may be a suitable option for patients who prioritize postoperative quality of life. However, the effectiveness of this approach requires further research to draw a definitive conclusion.

## Supporting information

S1 Checklist PRISMA 2020 checklist. (DOCX)

## Abbreviations

TLE: transanal local excision
TME: total mesorectal excision
LR: local recurrence
OS: overall survival
DFS: disease-free survival
LRFS: local recurrence-free survival
DSS: disease-specific survival
RCTs: randomized controlled trials
CSs: cohort studies
HR: hazard ratio
TEM: transanal endoscopic microsurgery
TAMIS: transanal minimally invasive surgery
RR: risk ratios
cCR: clinical complete remission
ncCR: near clinical complete remission
